# Supplementary material for: Transcriptomic Analysis of the CNL Gene Family in the Resistant Rice Cultivar IR28 in Response to Ustilaginoidea virens Infection
Source: Int J Mol Sci. 2024 Oct 3;25(19):10655. doi: 10.3390/ijms251910655 (PMC11477166; doi:10.3390/ijms251910655)
Supplement: Supplementary file 1 [file ijms-25-10655-s001.zip › ijms-3187116-supplementary.pdf]

Table S1. Mapping results of RNA-seq reads of resistant cultivar IR28 and susceptible cultivar WX98 and under *U. virens*-inoculated conditions

Library	Total paired-end raw reads (Gb)	Total paired-end clean reads (Gb)	Total mapped reads (%)[a]	Q20	Q30	Total mapped genes
IR28_1dpi_1	6.98	6.86	87.71	96.45	91.61	28795.00
IR28_1dpi_2	6.46	6.30	88.94	96.75	92.17	28840.00
IR28_1dpi_3	6.62	6.49	88.85	96.80	92.26	28653.00
IR28_5dpi_1	6.90	6.75	88.15	96.41	91.49	28714.00
IR28_5dpi_2	6.98	6.87	88.68	96.53	91.78	28488.00
IR28_5dpi_3	6.74	6.59	89.02	96.77	92.18	28630.00
IR28_9dpi_1	7.05	6.90	85.49	96.58	91.84	28789.00
IR28_9dpi_2	6.87	6.71	87.80	96.86	92.44	28627.00
IR28_9dpi_3	8.13	8.00	88.61	96.96	92.51	29017.00
IR28_13dpi_1	7.02	6.88	72.27	96.79	92.19	28175.00
IR28_13dpi_2	7.09	6.98	67.86	96.75	92.10	28331.00
IR28_13dpi_3	6.98	6.83	59.67	96.67	91.95	28067.00
WX98_1dpi_1	6.34	6.19	87.88	96.82	92.31	28656.00
WX98_1dpi_2	7.78	7.61	85.48	96.79	92.26	28790.00
WX98_1dpi_3	6.64	6.56	87.98	96.96	92.60	28801.00
WX98_5dpi_1	7.28	7.09	89.79	96.41	91.55	28728.00
WX98_5dpi_2	6.45	6.35	90.40	96.80	92.25	28650.00
WX98_5dpi_3	7.20	7.03	90.04	96.71	92.07	28719.00
WX98_9dpi_1	7.10	6.97	81.48	96.86	92.31	29344.00
WX98_9dpi_2	7.17	6.92	69.92	96.89	92.41	29378.00
WX98_9dpi_3	6.27	6.16	80.55	96.92	92.45	29784.00
WX98_13dpi_1	7.19	7.05	90.11	96.78	92.05	28307.00

WX98_13dpi_2	6.86	6.74	91.29	97.08	92.66	27683.00
WX98_13dpi_3	6.45	6.32	80.44	97.22	92.98	27599.00
Average	6.94	6.80	84.10	96.77	92.18	28648.54
Total	166.55	163.15				

[a]The percentages were calculated based on the total number of clean reads.

Table S2. DEGs Enriched KEGG pathways at 1, 5, 9, and 13 dpi in IR28.

Days post inoculation	KEGG ID	Description	Gene Ratio	Adjusted *P*-value
1	osa00940	Phenylpropanoid biosynthesis	86/616	3.46E-18
1	osa04626	Plant-pathogen interaction	57/616	3.43E-07
1	osa04016	MAPK signaling pathway - plant	36/616	0.002895
1	osa00520	Amino sugar and nucleotide sugar metabolism	36/616	0.003026
1	osa00592	alpha-Linolenic acid metabolism	20/616	0.004775
1	osa00941	Flavonoid biosynthesis	14/616	0.004775
1	osa00945	Stilbenoid, diarylheptanoid and gingerol biosynthesis	8/616	0.030074
1	osa04075	Plant hormone signal transduction	44/616	0.04481
1	osa00906	Carotenoid biosynthesis	11/616	0.047974
5	osa00196	Photosynthesis - antenna proteins	13/638	6.15E-08
5	osa00195	Photosynthesis	24/638	1.12E-07
5	osa00480	Glutathione metabolism	42/638	1.12E-07
5	osa00941	Flavonoid biosynthesis	20/638	9.40E-07
5	osa00940	Phenylpropanoid biosynthesis	63/638	9.40E-07
9	osa00940	Phenylpropanoid biosynthesis	101/932	9.66E-14
9	osa00040	Pentose and glucuronate interconversions	35/932	7.44E-06
9	osa00592	alpha-Linolenic acid metabolism	32/932	1.39E-05
9	osa04626	Plant-pathogen interaction	70/932	1.39E-05
9	osa00460	Cyanoamino acid metabolism	29/932	0.00017
9	osa00941	Flavonoid biosynthesis	20/932	0.000305
9	osa00906	Carotenoid biosynthesis	18/932	0.000718
9	osa00904	Diterpenoid biosynthesis	20/932	0.000809
9	osa00520	Amino sugar and nucleotide sugar metabolism	49/932	0.000809

9	osa01200	Carbon metabolism	87/932	0.000809
9	osa00430	Taurine and hypotaurine metabolism	9/932	0.000809
9	osa00630	Glyoxylate and dicarboxylate metabolism	33/932	0.004173
9	osa00710	Carbon fixation in photosynthetic organisms	30/932	0.004522
9	osa00410	beta-Alanine metabolism	21/932	0.005382
9	osa00480	Glutathione metabolism	39/932	0.00598
9	osa00591	Linoleic acid metabolism	9/932	0.008354
9	osa00500	Starch and sucrose metabolism	50/932	0.010954
9	osa00945	Stilbenoid, diarylheptanoid and gingerol biosynthesis	10/932	0.011858
9	osa00062	Fatty acid elongation	17/932	0.011912
9	osa00565	Ether lipid metabolism	13/932	0.011912
9	osa00944	Flavone and flavonol biosynthesis	7/932	0.011912
9	osa04016	MAPK signaling pathway - plant	43/932	0.013734
9	osa00905	Brassinosteroid biosynthesis	7/932	0.016329
9	osa00910	Nitrogen metabolism	15/932	0.038037
9	osa01040	Biosynthesis of unsaturated fatty acids	13/932	0.041069
9	osa04075	Plant hormone signal transduction	59/932	0.043395
9	osa00590	Arachidonic acid metabolism	9/932	0.052747
9	osa00561	Glycerolipid metabolism	24/932	0.069447
9	osa00260	Glycine, serine and threonine metabolism	23/932	0.088365
9	osa00564	Glycerophospholipid metabolism	29/932	0.124353
13	osa00196	Photosynthesis - antenna proteins	16/760	2.30E-11
13	osa00195	Photosynthesis	30/760	8.58E-11
13	osa00040	Pentose and glucuronate interconversions	35/760	2.19E-08
13	osa00940	Phenylpropanoid biosynthesis	64/760	0.000279
13	osa00910	Nitrogen metabolism	15/760	0.021034

13	osa00480	Glutathione metabolism	33/760	0.021034
13	osa00053	Ascorbate and aldarate metabolism	17/760	0.021034
13	osa00860	Porphyrin and chlorophyll metabolism	16/760	0.021034
13	osa00945	Stilbenoid, diarylheptanoid and gingerol biosynthesis	9/760	0.021926
13	osa00460	Cyanoamino acid metabolism	20/760	0.028437
13	osa00710	Carbon fixation in photosynthetic organisms	24/760	0.028741

Table S3. Primers used in this study.

Primer pairs	Sequence（5'to 3'）	Description
BGIOSGA028077-F	CATTACGAATCAAGAGATGTGG	qRT-PCR
BGIOSGA028077-R	GAGGTGCTGGATGCCGAA	qRT-PCR
BGIOSGA029433-F	GTGCCGTCGTGGTTTTAGT	qRT-PCR
BGIOSGA029433-R	GCTGTCTCGTTCAATGCG	qRT-PCR
BGIOSGA032258-F	AAGGATTTCACACACGAGCA	qRT-PCR
BGIOSGA032258-R	GCAATAACTTGGACAGCAGC	qRT-PCR
BGIOSGA032326-F	CGCACAGAAACCACCGAC	qRT-PCR
BGIOSGA032326-R	GAACCAACCGCTTGAAATC	qRT-PCR
BGIOSGA033963-F	CCAGAATCTTGGGCTCATACC	qRT-PCR
BGIOSGA033963-R	GGCACTGAAACTGTGAACTACC	qRT-PCR
BGIOSGA035581-F	ATCAATGGACCCTTTTCTGG	qRT-PCR
BGIOSGA035581-R	ATCAATGGACCCTTTTCTGG	qRT-PCR
BGIOSGA035675-F	GATTTGCTTTGTCACTTGCTTA	qRT-PCR
BGIOSGA035675-R	TCCTCTCCACCAGCCCTT	qRT-PCR
BGIOSGA036090-F	CTTATCCCAATCTTCAAAATGC	qRT-PCR
BGIOSGA036090-R	CTTCCAGGCACGATTGAGC	qRT-PCR
BGIOSGA036720-F	AAGTCGCCGAGGGATACCG	qRT-PCR
BGIOSGA036720-R	TCCAGTATCTTTGTATCTCGGG	qRT-PCR
BGIOSGA003230-F	CCAATAGTGGGATTTCTTTCAG	qRT-PCR
BGIOSGA003230-R	AGATTGGACTGTGGGTTAGAGA	qRT-PCR
BGIOSGA003329-F	GGGGTTTGGAGGTCTTGG	qRT-PCR
BGIOSGA003329-R	TGAAATCGGGCTTTTGTGA	qRT-PCR
BGIOSGA032859-F	GAGGCTCATCTTCTCAAAACC	qRT-PCR
BGIOSGA032859-R	ACCAGCAACAGCATCTTACC	qRT-PCR

OsBETV1-F	CTCATCCTCGACGGCTACTT	qRT-PCR
OsBETV1-R	ATCAGGAAGCAGCAATACGG	qRT-PCR
OsPR10b-F	CGGGCACCATCCACATCA	qRT-PCR
OsPR10b-R	CCAACACCTCAACCTTTAGCA	qRT-PCR
Ubiquitin10-F	TGGTCAGTAATCAGCCAGTTTGG	qRT-PCR
Ubiquitin10-R	GCACCACAAATACTTGACGAACAG	qRT-PCR

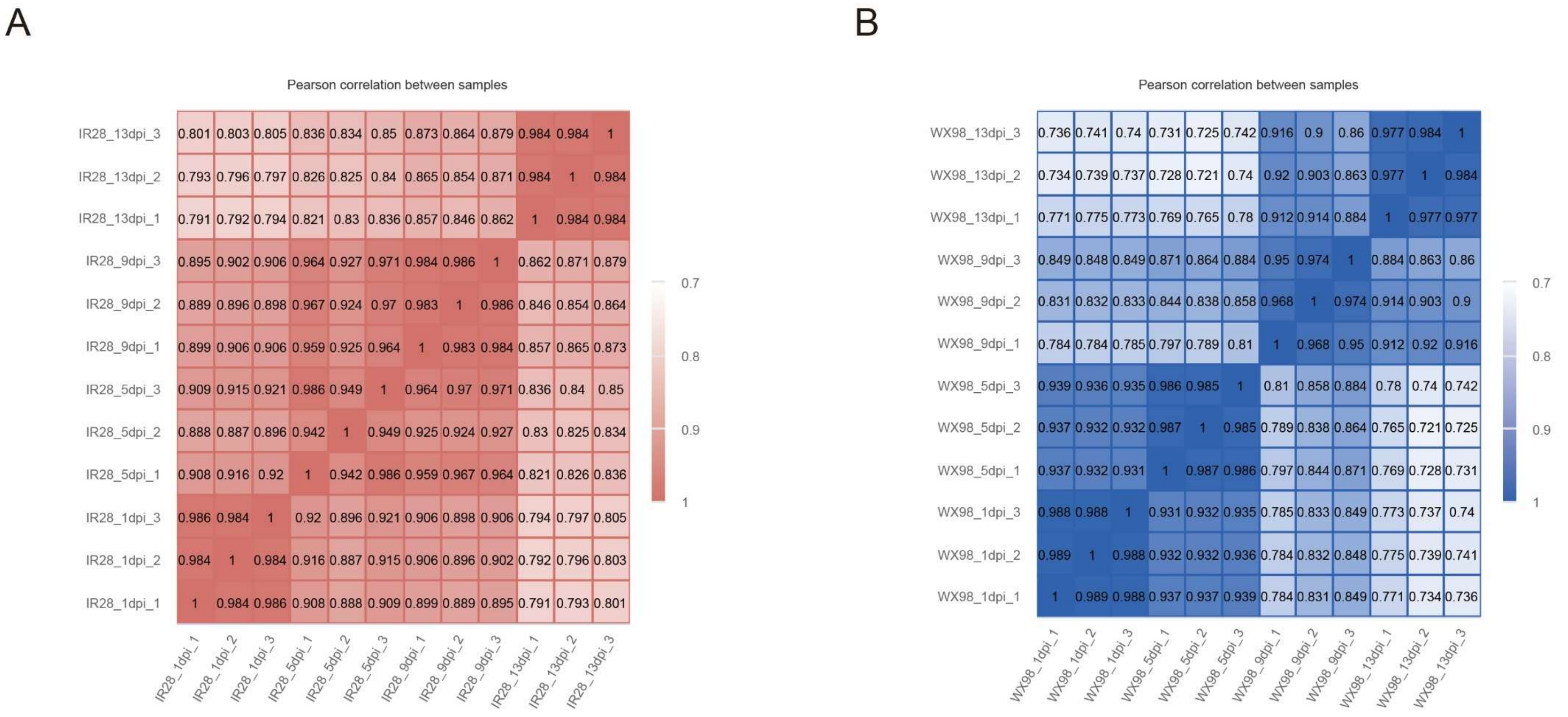

Figure S1. FPKM Pearson correlation coefficients of cultivar IR28 and WX98.

(A) FPKM Pearson correlation coefficients of cultivar IR28

(B) FPKM Pearson correlation coefficients of cultivar WX98
